# Supplementary material for: Are carbon emissions trading and green financial instruments synergistic? -Comprehensive quantitative research based on content analysis
Source: PLoS One. 2024 Mar 7;19(3):e0298601. doi: 10.1371/journal.pone.0298601 (PMC10919601; doi:10.1371/journal.pone.0298601)
Supplement: S1 Data — (ZIP) [file pone.0298601.s001.zip › Data/地级市Z_F工作报告.zip!/░▓╗╒/═¡┴Ω╩╨/└┤╫╘╣1⁄2╓┌║┼í╛┬φ┐╦╩2╛▌═°í┐_2010.docx]

各位代表：

　　现在，我代表市人民政府，向大会报告工作，请予审议，并请市政协委员和其他列席人员提出意见和建议。

　　一、2009年工作回顾

　　刚刚过去的2009年，是我们积极有效应对全球金融危机，实现经济社会持续平稳较快发展的一年。全市人民在省委、省政府和中共铜陵市委的坚强领导下，深入学习实践科学发展观，同心同德，奋力进取，扎实做好保增长、保民生、保稳定各项工作，较好地完成了市十四届人大三次会议确定的主要目标任务，发展势头明显好于预期。

　　预计，全市生产总值比上年增长14%左右。地方财政收入跨上30亿元新台阶，增长30 %。全社会固定资产投资280亿元，增长60%。社会消费品零售总额83亿元，增长18%。城镇居民人均可支配收入16765元，增长10.5%。农民人均纯收入6240元，增长12%，连续三年高于城镇居民人均可支配收入增幅。新增就业2.1万人，城镇登记失业率4.3%。万元GDP能耗下降4.3%，二氧化硫和化学需氧量排放量均超额完成省控制指标。人口出生率7.49‰，保持低生育水平。

　　过去的一年，我们以前所未有的力度抓投入，谋划并开工了一批事关长远发展的重大项目，投资总量相当于2006年的3倍，增长速度是1993年以来最快的一年；我们以前所未有的力度抓城乡大建设，城市新区建设和旧城改造如火如荼，城乡一体化发展全面展开；我们以前所未有的力度抓民生，提出了建设幸福铜陵的美好愿景，投入6亿元改善民生，是人民群众得到实惠最多的一年。与此同时，我们着力打造城市名片，发展循环经济成效显著，“铜都鉴日”圆满成功，先后荣获全国社会治安综合治理先进市、科技进步示范市、金融生态市等称号，铜陵在国内外的知名度进一步提升。我们可以自豪地说，铜陵经济社会发展已跨入新阶段，加速崛起已迈上新平台！

　　一年来，我们主要推进了以下几个方面的工作：

　　（一）创造性地落实国家和省应对金融危机的一揽子计划。我们紧密结合铜陵实际，把保企业作为保增长的重中之重，及时果断推出一揽子政策措施，全力帮助企业渡难关、上水平、增后劲。全年共兑现工业强市发展资金4400万元，为企业减免各类税费3.8亿元。全国首创的“金融超市”有效化解了中小企业融资难，全年金融机构新增贷款75亿元。完善“百名干部进百企” 帮扶长效机制等措施。以铜陵有色控股公司为代表的一大批企业不等不靠，应对危机的举措有声有色。工业经济发展逐季加快，全年规模以上工业增加值增长18%。

　　坚持打造千百亿工程。抓住国家实施产业调整振兴规划的机遇，及时组织编制铜产业、装备制造、化学工业等发展规划，同步谋划和跟进一批重大项目。铜陵有色投资60多亿元的铜冶炼工艺技术升级改造项目、上海中发电气投资20多亿元的特高压产业园项目已进入实施阶段，精达工业园等20个投资亿元以上的项目建成或投产，铜陵海螺日产1.2万吨水泥熟料生产线、皖能铜陵电厂100万千瓦机组等重大项目建设进展顺利。

　　推动服务业加快发展。积极落实国家鼓励汽车、家电下乡等政策措施，引导和鼓励普通商品房销售，促使汽车、住房消费大幅增长。浦发银行铜陵支行、交通银行铜陵分行相继开业，皖江农村合作银行顺利组建，铜都农村合作银行完成增资扩股，小额贷款公司实现一县三区全覆盖。华德物流配送中心开工建设，城山粮食储备加工物流等项目基本建成，中国铜产业网正式开通。雨润集团在我省兴建的最大的中央商务区落户铜陵并开工。江南文化园二期、金九五星级酒店等重点文化旅游业项目动工兴建。

　　（二）全力以赴抓招商引资和项目建设。我们把招商引资放在更加突出的位置。完善市领导联系重点项目制度，建立外来投资跨区域利益分成机制。精心组织参加第四届中部投资贸易博览会等大型招商活动，取得了一批重要招商成果。成功引进世纪华丰、福建祥兴、浙江盾安等一批国内外知名的大企业。全年实际利用内资196亿元、外资2.6亿美元，分别增长90%和30%。

　　认真谋划承接产业转移示范区建设。启动了江北产业园筹建工作，拉开了跨江发展的序幕。以战略思维和长远眼光，积极寻求与中央企业合作，对接签约项目12个，其中在安徽省与央企合作会议上集中签约10个，总投资达500多亿元。

　　加大资金争取和项目融资力度。通过三年的努力，争取国家批准我市为资源型城市转型试点城市。当年落实国家资源型城市转型试点市、可再生能源建筑应用示范市及狮子山区“金太阳”示范工程等国家和省各类补助资金12.3亿元，同口径增长31.3%，相当于全市地方财政收入的40%；市建投集团成功发行企业债券15亿元、融资50亿元；随着城市土地升值，全年实现土地出让收入20亿元，是上年的3倍。

　　完善项目投资工作推进机制。通过实施重点项目前期工作“以奖代补”、在建项目定期调度和及时督查等有效制度，全年完成项目建设拆迁面积63万平方米；开工和在建亿元以上项目84个，在建项目总数650个，投资总额达820亿元，增长51.9%。

　　（三）积极推进城乡大建设大发展。我们加速推进区域交通枢纽建设。铜陵火车站东站及站前广场投入运营；南京至安庆城际铁路建设顺利推进，铜陵长江公铁两用大桥已进入施工准备阶段；横港集装箱码头一期建成使用，铜陵港成为海峡两岸海运直航港口；由环城北路、滨江大道和沿新大道构成的城市外环道路全线拉开，城市交通枢纽工程西湖立交等基本建成。

　　大力开展市政设施建设与整治。北京路综合整治工程全面完成，天山大道、铜官大道整治基本完工，铜都大道三期、芜铜路、淮河路综合整治加快推进。西湖新区路网、“退田还湖”等进展顺利。结合大建设开展大拆违，全年拆除违章建筑10.3万平方米。计划整治的城市社区、天井湖亮化美化工程等如期完工，市容环境明显改善。

　　不断提升新农村建设水平。新增通乡公路 49公里、通乡公交线路3条。解决了3万多农村人口饮用水安全问题。新增农村沼气用户1700多户。完成了8座水库除险加固、130多公里农村河道清淤及东联圩与新民泵站技改等水利工程。在全国率先取消涉农行政性收费，累计落实和兑现各项惠农补贴4600多万元。培育“一村一品”示范村30个，新增市级以上农业产业化龙头企业10家，新建和改造规模化蔬菜基地1200亩，铜陵白姜地理标志保护产品获国家批准，国家现代林业示范市建设在全省率先启动。

　　着力推动县区和园区加快发展。整合市经济技术开发区和滨江循环经济工业试验园，园区主干路网和环境整治全面拉开。县区开发区配套功能得到加强。西湖、顺安和大通镇基础设施有所改善。市经济技术开发区完成生产总值占全市比重提高，县区生产总值占全市比重由上年的44%提高到50%。

　　（四）扎实推动改革攻坚和创新创业。我们成功引进战略投资者重组三佳集团和华源麻业，铜百集团、港务集团改制以及50家经营服务性事业单位转企改制基本完成。开展了市直部门“大部制”改革“回头看”，将城市具体行政执法和市容管理职能下划到辖区。在全省率先开展了公务用车货币化改革。制定了政府债务管理办法，建立起“借、用、还”良性机制。积极推进文化体制改革，全面启动了医药卫生体制改革。深化农村综合改革，完成新一轮乡镇机构改革。集体林权确权发证、农村宅基地登记发证工作基本完成，农村土地流转率由上年的19.6%提高到34.6%。

　　加强自主创新能力建设。与中国科技大学、合肥工业大学的合作进一步深入，与安徽师范大学正式建立全面合作关系。组织实施了染料敏化太阳能电池研发等30项产学研合作项目。新增省级工程技术研究中心3家，省级实验室实现“零”的突破，市生产力促进中心成为国家级示范中心。新增高新技术企业24家，高新技术产业增加值占规模以上工业增加值比重比上年提高2个百分点。铜陵县、铜官山区和郊区荣获全国科技进步考核先进县（区）称号。全年专利申请量突破550件，增长86%。建立了对中小企业专业技术人员购房补贴机制，人才引进和开发工作取得新成效。

　　积极促进全民创业。深化民营企业扶优扶强工作，全市有21家中小企业进入省级“专、精、特、新”重点调度企业行列，73家中小企业进入省级成长性小企业行列。深入开展火炬创业导师行动，成立了创业服务中心，开通了全民创业网，建成全省首家大学生创业培训基地和农民创业园一期工程。全年新注册民营企业1500家、个体工商户6000多户，分别增长52%和44%，非公经济实现增加值增长17.5%。

　　（五）大力发展循环经济促进节能减排。我们深入推进循环经济试点工作。铜冠冶化、铜化集团形成了循环工业组团，铜陵海螺开展工业固废利用，新亚星焦化一期等9个循环经济重点项目先后竣工或投入试生产，园区能源梯级利用和排放物循环利用积极推进。上峰水泥余热发电等20个项目先后开工建设。国家循环经济示范市建设总体规划通过专家评审。

　　推进生态环境建设“双十”工程。六国化工和冬瓜山矿废水循环利用、环天井湖和玉带河污水管网等工程投入运行，西湖污水处理厂建成，城北污水处理厂主体工程完工。皖能铜陵电厂3号机组、富鑫钢铁等烟气脱硫工程点火运行。铜陵县滨江化工区企业关停、转移取得重大进展。淘汰24户企业落后产能。铜陵淡水豚保护区湿地保护工程全面完工。我市循环经济和节能减排工作得到了吴邦国等党和国家领导人的肯定。

　　（六）不断创新以改善民生为重点的社会建设。我们坚持以创业带动就业。全面启动就业“1+2”工程，通过减免、缓缴、核销、补贴社会保险费与奖励等综合措施，千方百计稳定企业现有岗位，帮助8700多名下岗失业人员实现再就业，近2万人接受了职业技能培训，城镇“零就业家庭”做到了动态消除。

　　社会保障体系进一步完善。城镇居民医疗保险基本实现全覆盖，保障水平进一步提高。新型农村社会养老保险制度在全省率先实施，参保人数达6.6万人，铜陵县成为国家首批试点县。新型农村合作医疗年人均筹资标准上调至130元。城乡低保、城镇未参保集体企业退休人员基本生活保障进一步扩面提标。率先建立了城乡低收入群体和特困群众临时救助制度，200多位贫困白内障患者免费通过手术重现光明。制定并实施了全市棚户区三年改造计划，改造棚户区面积25万平方米、竣工10万平方米，新建廉租住房2700多套，有6900多户困难家庭领取廉租住房租赁补贴，保障性住房分配管理更加规范、透明、公正。

　　各项社会事业取得新发展。中小学校舍安全改造工程在全省率先启动，铜陵职业技术学院新校区、中等职业教育园区建设全面铺开。城乡卫生、计生服务机构规范化建设完成目标任务，公共卫生服务、社区医疗服务均等化走在全省前列。市人民医院新门诊大楼等动工兴建。甲型H1N1流感防控工作取得阶段性胜利，胥坝乡血吸虫病传染源控制综合治理全面完成。创建国家卫生城市工作扎实推进。铜博物馆新馆开工建设，文化信息资源共享、广播电视村村通、乡镇综合文化站、农家书屋等惠民工程实现建设目标，铜陵县被评为全国文化先进单位。成功举办市第一届全民健身运动会。高质量地完成了第二次经济普查工作。

　　（七）精心维护社会大局和谐稳定。我们加强了社会治安综合治理。治安防控体系不断完善，全市刑事案件发案率连年下降，人民群众的安全感进一步增强。开展“四大、四进、四送”活动，从源头上有效化解了矛盾纠纷。法制宣传教育、行政复议、人民调解作用得到加强和发挥，集中解决了一批群众反映的实际问题。组织开展了安全生产年活动，强化安全生产责任制落实和各类安全隐患的排查整治，消除了一批重大安全隐患，全年安全生产事故次数、死亡人数分别下降23.1%和15.9%，保持了社会大局和谐稳定。

　　深入推进和谐社会建设。我们满怀豪情地组织开展了新中国成立60周年系列庆祝活动。大力建设“四优”和谐社区，铜官山区成为全国首批和谐社区建设示范城区。积极支持工会、共青团、妇联、科协等人民团体参与社会管理和公共服务。大力支持驻铜部队建设，争创新一轮全省双拥模范城工作取得积极进展，国防动员、民兵预备役、人民防空等建设水平得到提高。国家安全、对台和民族宗教等工作得到加强。统筹做好外事、侨务和外宣等工作，“走进铜陵”系列专题片在中央电视台连续播出，我市对外影响力进一步提高。

　　各位代表！过去的一年，也是政府自身建设得到加强、施政水平经受考验、推动科学发展能力不断提高的一年。我们认真开展深入学习实践科学发展观活动，进一步完善有利于促进科学发展的体制机制。坚持重大事项向市人大常委会报告和向市政协通报制度，自觉接受监督，按时办结市人大代表建议187件、市政协提案261件。启动依法行政示范市建设工作，全面推行行政处罚（决定）自由裁量权基准制度。市级行政审批项目由258项削减至188项，进入政务服务中心窗口项目增长32.9%，累计办理审批服务事项60多万件，增长20%。建成全市党政机关协同办公网，开通了“12345”政务服务热线，增强了政府信息公开网、市民论坛、行风热线等载体功能，进一步拓展了服务群众的渠道。全面推行行政首长问责制，在全省率先实施机关效能投诉追究制。加强节约型机关建设，开展清理“小金库”工作，行政经费支出实现“五个零增长”。出台了政府投资项目管理、财政资金使用和规范建设工程、政府采购招投标等管理制度，推行了政府性项目投资和市直部门财务“两个审计全覆盖”，开展了工程建设领域突出问题专项治理，惩治和预防腐败体系不断完善。

　　各位代表！过去的一年，我们能在极其困难复杂的条件下，取得经济社会发展的优异成绩，实属来之不易，必须倍加珍惜！这是省委、省政府和中共铜陵市委正确领导的结果，是全市人民同心同德、顽强拼搏的结果，是社会方方面面关心帮助、鼎力支持的结果。在这里，我代表市人民政府，向全市各族人民致以崇高的敬意！向人大代表、政协委员，向各民主党派、工商联、各人民团体、无党派人士、离退休老同志和社会各界人士，向驻铜人民解放军、武警官兵和政法干警，向所有关心支持铜陵发展的省直各部门、兄弟市和海内外友好人士，向在我市创业的境内外投资者、建设者，表示衷心的感谢！

　　在充分肯定成绩的同时，我们也清醒地看到，当前经济回升的基础还不稳定、不巩固、不平衡，影响经济发展的不利因素依然存在。我市经济社会发展中仍面临一些突出矛盾和问题，主要是：经济总量仍然偏小；主导产业链条延伸不够，服务业发展仍显不足；环境容量压力大，节能减排任务仍十分艰巨；城市基础设施历史欠账较多、管理上还有不少薄弱环节；政府工作还有不少需要改进和提高的地方，少数政府工作人员和领导干部服务意识不强、工作作风不实、为政不勤不廉等问题也还不同程度存在。对此，我们一定要高度重视，采取切实有效措施认真加以解决。

　　二、2010年主要工作

　　今年是“十一五”最后一年，也是谋划“十二五”的关键之年。从宏观形势看，国家仍将实施积极的财政政策和适度宽松的货币政策，特别是推进中部崛起、实施产业振兴、促进资源型城市转型、建设皖江城市带承接产业转移示范区等政策叠加效应，将为我市提供难得的发展机遇。从我市来看，经过历届市委、市政府坚持不懈地努力，全市上下加快发展意识不断增强，发展理念不断更新，发展思路更加清晰，发展内生动力与物质技术基础持续增强，加之市场回暖、主导产品价格回升等，我市具备了诸多发展的有利条件。当然，也要看到，当前国际金融危机余波未尽，经济运行尚未根本好转，形势的复杂性不容低估；我市经济社会发展中累积的结构性、体制性矛盾尚未根本解决。同时区域发展的竞争日益激烈。我们一定要审时度势，既增强机遇意识，又增强忧患意识，振奋精神，努力工作，不断推动我市经济社会更好更快地发展。

　　今年工作的总体要求是：以邓小平理论和“三个代表”重要思想为指导，深入贯彻落实科学发展观，按照省委、省政府和市委的总体部署，加快转变经济发展方式，增加总量，优化结构，惠及民生，提升效能，全面完成“十一五”规划目标，为提前八年全面建成小康社会和建设幸福铜陵奠定更加坚实的基础。

　　综合考虑各种因素，今年经济社会发展主要预期目标是：全市生产总值增长13%以上（突破400亿元）；地方财政收入增幅高于地区生产总值增幅（财政总收入突破80亿元）；社会消费品零售总额增长20%以上；全社会固定资产投资增长25%以上；城镇居民人均可支配收入和农民人均纯收入分别增长10%和11%以上；新增就业2万人，城镇登记失业率控制在4.3%以内；万元GDP能耗下降4.2%，二氧化硫和化学需氧量排放量完成省控制目标；人口自然增长率控制在6.5‰以内。

　　我们将重点做好以下八个方面工作：

　　（一）着力扩大有效需求，保持经济持续较快增长。坚持以大投入带动大发展，增加投资总量。开展铁路建设攻坚年、央企合作项目落地年、跨江发展突破年等重大活动，统筹安排投资1000万元以上重点项目超200个、亿元以上重点项目超100个，在建项目数争取突破1000个，带动全社会固定资产投资350亿元以上。加快建设铜陵有色铜冶炼工艺技术升级改造、铜陵长江公铁两用大桥等工程，推进铜化集团大合成氨等一批投资20亿元以上的重大项目，谋划和储备一批投资超50亿元、甚至超100亿元的项目。健全重大项目调度、重点项目领导分工联系制度，提高并联审批速度，切实解决征地拆迁难等问题，促进项目快落地、快建设、早投产。

　　壮大工业经济规模。将企业减负、贷款担保等已出台的政策措施延长一年，并进一步支持企业开展资源控制、资产重组、技术改造和名牌培育等工作。继续完善“金融超市”等银政、银企合作机制，推动铜陵有色财务公司早日开业，新增小额贷款公司和村镇银行3—5家，全市金融机构贷款余额争取超过400亿元。帮助全威铜业、锐展科技等一批骨干企业实现达产达效，支持皖能铜陵电厂100万千瓦机组、铜陵海螺日产1.2万吨水泥熟料生产线、华纳万吨铜箔等一批项目建成投产。力争全市工业企业主营业务收入突破1000亿元。

　　提升消费拉动作用。积极落实国家鼓励汽车、家电下乡等扩大消费政策，完善城乡市场流通网络，不断优化消费环境，力争全市社会消费品零售总额突破100亿元。认真落实外贸促进政策，积极申报铜陵电子材料出口基地，加快B型公用型保税物流中心建设，充分发挥进出口孵化促进中心作用，支持优势骨干企业开展境外资源开发和企业并购。力争全年外贸进出口总额增长25%以上。

　　大力发展都市型农业。以推进农业产业化为重点，加大生姜、凤丹龙头企业培育力度，全年新增省和市级龙头企业10家以上。抓好农业园区（基地）标准化提质工作，新建标准化生姜基地1100亩、蔬菜基地2000亩、养殖水面1000亩、畜禽养殖小区10个。开展农产品品牌争创工作，继续培育“一村一品”，新增无公害农产品、绿色食品和有机食品10件以上。认真落实惠农补贴政策，促进农业提效、农民增收。

　　（二）着力加大招商引资力度，建好承接产业转移示范区。进一步营造“大招商、招大商”氛围，推动招商引资实现新突破。完善招商引资目标考核和政策激励机制，不断创新招商方式，提高招商实效。充分利用我市岸线资源、产业基础和配套条件，加强与世界500强、行业龙头企业和领军人物合作。继续创造条件，不断扩大与中央和省属企业合作成果，争取合同项目早日落地，协议项目转成合同，意向项目转成协议，在谈项目早日签约。力争全年实际利用内资255亿元、增长30%，利用外资2.9亿美元、增长10%以上。

　　推进承接产业转移取得新成果。积极参与泛长三角区域发展分工合作。全面启动江北产业园核心区供水、供电、道路等基础设施建设，争取早日具备承接产业转移条件。推动市经济技术开发区和县区开发区加快配套功能建设，进一步提升项目落地能力。大力吸引长三角等地区来铜兴办与我市产业关联度强的“异地产业园”或产业集中区。加快特种金属材料等特色产业园区建设，引导各园区错位竞争、集群化发展。筹建铜陵长江公铁两用大桥南岸产业和物流园区，促进港口及临港区域整体开发和建设。

　　（三）着力调整优化产业结构，加速资源型城市转型步伐。以打造千百亿工程为抓手，加快改造提升传统优势产业，调整产业产品结构。开发铜加工系列产品，提升铜材加工规模和水平，进一步促进铜加工、铜贸易、铜文化融合发展。加强与中国石油、中国化工、中国盐业进行战略性合作，发展大化工、盐化工、氟化工。大力发展矿山机械、环保设备、高压阀门等产品，壮大装备制造业。加快开发麻纺系列产品，积极引进和培育服装品牌，增强纺织服装业竞争力。

　　积极发展战略性新兴产业。坚持市场导向原则，充分发挥现有产业基础和潜力，借助中科大、合工大、中科院合肥分院等合作方的技术优势，着力培育电子信息、铜基新材料、节能环保、新能源、光电等产业。以印制电路板产业园、光电产业园建设为载体，大力引进战略投资者，加快建设压延铜箔、“金太阳”工程、锂电池隔膜、芯片制造与封装等一批重点项目，打造国家级铜基电子材料产业和光电产业基地。

　　推动服务业实现跨越式发展。进一步加大政策支持和落实力度，加快发展一批品牌商贸物流企业，实施步行街综合改造等重点商业组团项目，新建2—3家区域性商场，争取开工建设中部地区成品油和化工产品物流基地、皖能煤炭运输集散基地，发展大物流、大商贸。加快发展文化旅游业，搞好工业旅游开发，提升休闲旅游产品档次。争取再建设1—2家五星级酒店。开工建设会展中心，发展商务会展经济。加快发展信息咨询、创意设计、服务外包等产业，促进信息产业与服务业融合发展。

　　（四）着力推动改革创新，增强发展内在活力。坚持深化重点领域和关键环节改革。加强国有资产监管，健全运营风险防范机制，提升资本运营效率。鼓励各类企业扩大直接融资规模，争取再发行企业债券15亿元以上，扶持1—2家企业上市。继续深化行政管理体制改革，建立与城市化发展相适应的城市管理体制，完成县区机构改革、事业单位岗位设置和绩效工资改革任务。加快推进医药卫生体制改革，重点是基层医药卫生体制综合改革。完善公务用车改革。基本完成文化行政管理体制改革任务。重点做好放宽户籍制度和村改居工作，加快城市化进程。

　　提升自主创新能力。进一步扩大与高等院校和科研院所的合作，争取创建1—2个国家级研究机构或研发平台。通过设立成果转化资金，加速科技成果转化。增强中科大创业园孵化功能，建立入园项目动态管理机制。推进全国知识产权示范市建设，争取建设国家铜产业专利产业化试点基地。支持企业提高产品质量和技术水平，争创5—10个国家和省级名牌。实施人才强市战略，筹建人才公寓，对高层次人才来铜工作给予项目经费资助、住房和生活补贴。

　　大力发展非公经济。完善和落实发展非公经济的政策措施，加大扶优扶强力度。加快大学生创业园、农民工创业园等创业平台建设。设立高校毕业生创业引导扶持资金，健全创业者融资担保制度，向所有符合条件的创业者提供小额贷款担保。进一步拓宽民间资本的发展空间，积极开展创业指导服务，激励更多的人投身创业大潮。力争新增个体工商户5000户、民营企业1500户以上。

　　（五）着力推进城乡大建设，加快城乡一体化发展。新区大建设要有新成果。基本建成西湖新区路网主干道路，再开工一批标志性工程；基本拉开东部城区起步区路网框架，开工建设政务文化新区；全面实施南部城区骨干路网改造工程，基本建成政务服务中心各主体工程框架。加快新区之间快速通道建设进度，正式打通城市外环路。将环城北路向东部城区延伸、滨江大道向南部城区延伸、学士路向狮子山老城区延伸、湖东路向市开发区延伸。积极谋划市区到江心洲的跨江通道建设。

　　老城区改造要有新形象。开工建设长江东路接沿新大道隧道、北京路下穿接市行政中心工程、俞家村跨铁路立交桥等重点项目，完成淮河大道综合整治工程，建成老铜百地下通道、家得利人行天桥，规划建设长江二路，进一步缓解城区交通压力。实施停车场建设规划，新建一批货运及小车停车场，有效缓解停车难问题。完成27个社区综合整治任务。继续实施支次道路改造工程。综合治理黑沙河下游等排水管网，提高排涝能力。推进植物园、螺蛳山、笔架山和鹞山公园综合整治工程，开工建设滨江世纪广场及生活岸线项目，让广大市民能在此与江水“零距离”接触。

　　新农村建设要有新进展。以土地整治为抓手，以中心村建设为重点，整合各级各类支农惠农资金，认真落实建设规划，统筹建设农村基础设施，整村推进新农村建设。全面启动农村电网第二轮升级改造，实施农村清洁工程，完成农村185公里河道清淤任务，推进长江铜陵段（南夹江）综合治理项目前期工作。落实加快县区发展的政策措施，开展城乡结对帮扶，增强县区自主发展能力。

　　（六）着力推进节能减排，提高城乡环境质量。我们既要金山银山，更要绿水青山。加快节能减排重点项目建设，组织实施冶炼、化工、建材等行业污染治理再提高工程，按期完成铜陵县滨江化工区企业关闭和转移工作。深入开展运输泼洒和建筑施工扬尘整治，坚决遏制可吸入颗粒物污染。大力推进南部城区粉尘治理，限期关停小采石场等污染环境的小企业。确保城市生活垃圾无害化处理工程投入运行，结束建市以来生活垃圾简易填埋的历史。加强矿山生态保护和土地复垦整理，积极推进新桥河、狼尾湖环境综合整治。严查重处环境违法案件，全面启动环保模范城市创建工作，加快建设资源节约型和环境友好型社会。

　　进一步推动循环经济发展。建成滨江循环经济工业试验园道路及铁路专用线，启动横港循环经济工业示范区基础设施建设，推进城山农业循环经济试验区建设。通过园区内各种管线连接，实现企业间能源梯级利用、排放物循环利用，完善循环经济产业链。抓紧制定低碳经济发展方案，继续推进一批循环经济重点项目，争取突破一批关键技术，推进国家循环经济试点市向示范市转变。加快推进国家现代林业示范市建设，认真实施城市绿化三年规划和农村村庄绿化工程，进一步绿化、美化铜陵。

　　（七）着力开展文明创建活动，促进社会和谐稳定。深入推进社会主义核心价值体系建设，加强未成年人思想道德教育。广泛开展群众性文化和全民健身活动，进一步提升全民科学文化素质。继续推进“五五”普法教育，开展法律援助活动。发挥工会、共青团、妇联、科协等人民团体的桥梁纽带作用，鼓励开展社会救助和义工活动，动员全社会力量参与社会管理和公共服务。深入开展国防教育，支持驻铜解放军和武警部队建设，不断提高预备役部队、民兵和人民防空建设水平。积极开展双拥共建活动，再创全国双拥模范城。大力开展城乡爱国卫生运动，实现国家卫生城市创建目标。加大城市管理力度，坚持重心下移、事权下放原则，进一步理顺和规范市、区两级城市建设管理体制，加大市容环境、交通秩序等重点领域整治力度，深入开展查违拆违活动。实施城市社区提升工程，力争90%的社区达到“四优”和谐社区标准。

　　加强平安铜陵建设。进一步加强综治维稳工作基层基础建设，加大创建平安铜陵技防建设力度，切实加强以“打黑除恶”为龙头的严打整治斗争，积极开展“长安杯”创建活动，进一步增强人民群众的安全感。深入开展“四大、四进、四送”活动，最大限度地增加和谐因素、减少不和谐因素。牢固树立安全发展理念，强化落实安全生产责任制，完善安全生产科学管理与监督体系，深化重点行业领域专项整治，坚决杜绝重特大安全事故的发生。建立完善食品药品监管长效机制，严把准入和质量关，保障全市人民饮食安全和身体健康。加快公共安全应急体系建设，切实增强公共卫生、突发灾害的应急反应能力。

　　（八）着力提升民生工程，统筹发展社会事业。今年，我们将在前两年实施民生工程的基础上，优化项目，扩面提标，政府投入资金8亿元以上，实施48项民生工程，让全市人民得到更多实惠，进一步增强幸福感。

　　积极扩大就业。加大就业专项资金的投入，落实更加积极的就业政策。深入开展就业援助活动，健全面向所有劳动者的职业技能培训补贴制度，就业信息网络向街道、社区延伸。扎实做好高校毕业生、农民工、下岗失业人员、残疾人以及失地农民等群体的就业帮扶工作。

　　进一步健全社会保障体系。继续加大各项保险扩面征缴力度，扩大新型农村养老保险覆盖面，力争实现城乡养老保险全覆盖。调整完善各项医疗保险政策，建立特大疾病医疗费用统筹制度，将新型农村合作医疗筹资标准提高至180元。坚持以尊老、爱幼、助残为重点，健全城乡社会救助制度。适时提高城乡低保、困难救助等保障标准，积极发展社会福利、红十字和各项慈善事业，争创全国残疾人工作示范城市。

　　加大棚户区改造力度。今年是全市棚户区改造攻坚之年，我们将加强协调、全力推进，对纳入棚户区改造计划的项目，在土地出让金返还、市政基础设施配套等方面给予最大的支持。同时，继续推进经济适用房和安置房建设，鼓励困难企业集资建房，全年棚户区改造、廉租住房等施工面积120万平方米以上。全面落实保障性住房计划，对所有符合条件的城镇低收入住房困难家庭发放廉租住房租赁补贴。

　　坚持公交优先。继续优化公交线路，增加公交车辆，公交客运通达江心洲以外的所有乡镇。实施洲圩区水上交通安全工程，新建改造渡口码头及配套设施，添置渡船，切实改善洲区群众的出行条件。对全市出租汽车进行全面更新。

　　推动均衡教育向深度和广度拓展。新建改造中小学校舍32.4万平方米，把全市中小学建成安全最可靠、家长最放心的地方。实施学前教育标准化建设和规范化管理，对符合条件的幼儿园实行“以奖代补”。对全市普通高中家庭经济困难学生进行资助。整合中等职业教育资源，积极筹建高级技师学院，建成铜陵职业技术学院新校区，全力打造皖中南职业教育基地。

　　提高公共卫生均等化服务水平。在全市基层医疗机构实行基本药物零差率销售，让广大市民享受医药卫生体制改革成果。继续推进乡镇卫生院和村卫生室、社区卫生服务中心和服务站等基层卫生服务体系标准化建设，加快市人民医院门诊医技大楼等重点医疗设施建设进度，全面改善城乡公共卫生服务条件。

　　加快发展文化等社会事业。加快铜博物馆新馆建设进度并积极申报中国铜文化博物馆；全面启动建设新广播电视中心和报业大楼、体育中心、图书馆及档案馆新馆等工程；继续推进乡镇、村和社区公共文化服务设施建设。办好第十一届中国（铜陵）青铜文化博览会、省首届民俗文化节和国际铜雕塑大赛。加强统计监测，开展第六次全国人口普查工作。健全计划生育利益导向机制，稳定低生育水平。继续加强外事、侨务、民族、宗教、对台、气象、地震、地方志、档案等工作。

　　在做好上述工作的同时，我们将立足当前，着眼长远，围绕力争实现地区生产总值、地方财政收入“两个翻番”的目标，认真组织编制好“十二五”规划。

　　三、加强政府自身建设

　　面对复杂的经济形势、繁重的发展任务，政府责任重大、使命在肩，我们必须进一步转变政府职能，全面提高行政效能，为加快发展创造更加优良的政务服务环境。

　　加强学习，增强本领。各级政府公务员要加强学习，深入调查研究，认清深刻变化的发展形势，把握科学发展的内在规律，不断增强推动科学发展的能力和水平。要切实转变观念，敢于突破常规，善于求新思变，该放的事放得开，该管的事管得住，该干的事干得好，该抓的事抓得实。要增强公仆意识，多为群众办实事、办好事，多做打基础、管长远的工作。

　　依法行政，规范公开。各级政府要自觉接受人大法律监督和政协民主监督，认真办理人大议案、代表建议和政协提案，充分听取各民主党派、工商联、无党派人士及各人民团体的意见，广泛接受社会公众和新闻舆论监督。建立健全决策咨询、公示、听证等制度，促进民主、科学决策。编制市直部门管理权限目录，细化自由裁量权的标准、层级和行使规则，并从今年起一律向社会公开，未经公开的一律不得行使。推进规范行政执法行为示范市建设，认真清理行政执法事项，逐项绘制行政职权流程图，规范执法程序和行为。完善政府信息公开运行机制，推进机关事务公开工作，建立规划审批、财政预算、政府投资等信息发布制度，切实让权力在阳光下运行。

　　改进作风，狠抓落实。各级政府要大力倡导紧张快干、雷厉风行的作风，增强服务至上、效率优先的理念，形成相互支持、通力配合的局面。要进一步精简行政审批，从今年起，实行行政许可和行政审批事项年度清理和公告制度；市直部门行政许可、审批事项除经市政府批准外，一律进入政务服务中心办理，凡进入政务服务中心办理的有关事项，不得再要求相对人到部门办理相关手续；取消市级设立的所有行政事业性收费项目，对国家和省制定的行政事业性收费，凡有下限标准的一律按下限收取，并实行年度公布制度。继续推行项目代理审批、一单收费、一窗受理、限期办结等高效服务方式。建立健全效能建设监督的长效机制，完善目标管理办法，严肃效能追究，促进服务提速提效。

　　廉洁奉公，取信于民。各级政府要严格落实党风廉政建设责任制，推进惩治和预防腐败体系建设，建立以制度管事、理财的长效机制，切实开展财政“基础管理年”活动，着力推进节约型机关建设，加强审计和政纪监督，促进廉洁从政。政府公务员都要牢固树立立党为公、执政为民的理念，食人民俸禄、为人民办事；都要牢固确立科学发展观和正确的政绩观，牢记使命、信守承诺，加强沟通、促进团结，坚持原则、和谐共融，聚精会神搞建设，一门心思促发展，做出经得起历史、经得起人民检验的成绩。

　　各位代表！铜陵的发展已进入一个非常重要的时期。只要我们凝心聚力、勇于拼搏，就一定能圆满完成“十一五”规划确定的各项任务，就一定能开创率先发展、科学发展、和谐发展的新局面，任何困难和挑战都阻挡不了我们前进的步伐！让我们高举中国特色社会主义伟大旗帜，深入贯彻落实科学发展观，在省委、省政府和中共铜陵市委的坚强领导下，脚踏实地，扎实工作，为实现提前八年全面建成小康社会和建设幸福铜陵的宏伟目标而努力奋斗！
